# Survival and causes of death among released brown hares (*Lepus europaeus* Pallas, 1778) in Central Poland

**DOI:** 10.1007/s13364-012-0081-1

**Published:** 2012-05-02

**Authors:** Magdalena Misiorowska, Michał Wasilewski

**Affiliations:** Department of Forest Zoology and Wildlife Management, Faculty of Forestry, Warsaw University of Life Sciences-SGGW, ul. Nowoursynowska 159, 02-776 Warsaw, Poland

**Keywords:** Brown hare, *Lepus europaeus*, Radiotelemetry, Survival, Mortality

## Abstract

We describe the results of our research on population dynamics among brown hares reared in enclosures and then released into suitable natural habitat. Radio-tracking was used to follow the fate of 60 released brown hares over a 4-year period, extending between November 2005 and November 2009. The survival rate among these animals after 12 months was estimated to be 37 %, with 22 tagged individuals surviving beyond 1 year post-release. The highest (40 %) level of mortality characterised the first month after release, while a second period of enhanced mortality coincided with the breeding season (altogether accounting for a 20 % mortality rate). There was no significant relationship between body mass and mortality rate in the first month following release. A natural cause of death was predation by mammals, which accounted for some 31 % of all losses. Remaining causes were poaching (13 %), hits by vehicles (7 %) and unidentified causes (9 %). However, in at least 40 % of cases, it was not possible to determine the date when a released animal died, to say nothing of the cause of death.

## Introduction

Since the mid 1960s, the population of brown hares across Europe has declined to such an extent that the species is now deemed under serious threat (Ninov 1990; Slamečka 1991; Marboutin and Peroux 1995; Tapper 1995; Hutchings and Harris 1996; Slamečka et al. 1997; Edwards et al. 2000; Marboutin et al. 2003; Vaughan et al. 2003; Jezierski 2004; Schmidt et al. 2004; Smith et al. 2005; Santilli and Galardi 2006; Vuković and Tvrtković 2006; Roedenbeck and Voser 2008). The declines in numbers of hares noted in Norway, Germany, Austria and Switzerland have, in turn, led to the species being Red-Listed in those countries as “Near Threatened” or “Threatened” (Boye 1996; Pfister et al. 2002; Reichline et al. 2006; Roedenbeck and Voser 2008). Similarly, in Poland, which was once renowned for its very large population of the species, the situation is also starting to become worrisome.

There are many factors considered as responsible for the decline in the number of brown hares. Among these, agricultural intensification — increasing use of chemicals, changes in crop structure (monocultures) and agrotechnical works as well as changes in the environment resulting in a loss of biodiversity (e.g., changes in the landscape — reduction of hedges and path of mid-field woodlots), can significantly affect the biological diversity of ecosystems inhabited by hares. Increases in numbers of predators, disease and climate change are additional factors which may have negative impact on hare local populations (Kałuziński and Pielowski 1976; Rattenborg 1991; Slamečka 1991; Lewandowski and Nowakowski 1993; Marboutin and Peroux 1995; Reynolds and Tapper 1995a; Slamečka et al.1997; Homolka and Zima 1999; Panek and Kamieniarz 1999; Dziedzic et al. 2000; Edwards et al. 2000; Frolich et al. 2003; Vaughan et al. 2003; Schmidt et al. 2004; Smith et al. 2005; Van Wieren et al. 2006; Roedenbeck and Voser 2008).

In many European countries, diverse steps have been taken to try and save, or — even more ambitiously — rebuild the steadily declining populations of brown hares. In Poland, action seeking to protect hares (e.g., reductions in predator populations, and cessation of hunting in districts where population densities have fallen to around 5 animals/km^2^), and enhance their living conditions (e.g., entailed diversification of habitat) are being taken. Actions have also been made to release hares obtained via various kinds of captive breeding in cages or enclosures directed to restocking the declining local hare population. Measures of this kind are considered capable of playing a major role in the protection of animal species, inasmuch as they may increase levels of genetic diversity and encourage a return of endangered populations to a state of demographic balance. This, in turn, raises their chances of survival. However, there are no available data addressing the population dynamics of hares originating in captivity, and then released into the natural environment in Poland, and only a few similar studies have been conducted in other European countries.

We present the results of the first Polish study assessing survival and causes of death of hares originating in captivity and subsequently released into the natural environment for restocking local population.

### Study area

Studies was carried out over a 4-year period (November 2005–November 2009), in Central Poland, and specifically in the community of Maciejowice (51 °45′N, 21 °26′E). This study area, located around 60 km south of Warsaw, was only delineated more precisely following the actual release of hares, by reference to the most distant radiolocations obtained in practice for the animals. The area obtained in this way covers some 18 km^2^, the western boundary being set by the River Vistula, the northern and eastern by forest, and the southern by the River Bończycha. The eastern, western and northern parts of the area have floodbanks separating areas under active management from a broad belt of floodlands between embankments, as well as the Rivers Vistula and Bończycha.

More than half (ca. 53 %) of the study area is covered by cultivated fields. Windbreaks, hedges and areas of planted trees in the middle of fields together account for some 10 % of the area, while the land within floodbanks accounts for 8 %. The forest within the study area (representing some 18 % of it) forms part of a larger complex. Orchards and meadows in turn cover ca. 3 % of the area, while areas that hares cannot make use of at all (e.g., roads, built-up areas and rivers) account for ca. 8 %. (For a detailed description of the study area, see Misiorowska and Wasilewski 2008.)

Hares were present in the study area. The pre-release density of hares was low, approx. 10 individuals/km^2^. A very similar pattern of hare density was noted in the surrounding area. The carnivores present are the red fox (*Vulpes vulpes*), raccoon-dog (*Nyctereutes procyonoides*), beech marten (*Martes foina*) and pine marten (*Marten martes*). It is also obviously possible for the hares to encounter dogs (*Canis familiaris*) and cats (*Felis catus*) in the area, which penetrate the fields and forest both day and night, leaving any human owners and carers behind.

## Materials and methods

The material for study comprised 78 hares raised in a 20-ha open-field enclosures within Świebodzin Forest District, and then released in the study area in batches of 30, 30 and 18 individuals, in 2005, 2006 and 2007, respectively (Table 1). All animals were born in open-field enclosures, but their ancestors were captured from the wilds. The age of released hares was at least 6 months. The releases took place in autumn (late November), all animals being weighed and sexed prior to the fitting of transmitters in 60 cases. The mean weigh of females was 3.7 kg (range: 2.7–4.9) and that of the males was 3.8 kg (range: 2.7–4.8). The number of radio-collared hares was 29, 15 and 16 in 2005, 2006 and 2007, respectively.

**Table 1:** A characterisation of the hares released in successive years in Central Poland

	Year			Total
	November 2005	November 2006	November 2007	
Females	18	11	10	39
Males	11	4	6	21
All	29 (30)	15 (30)	16 (18)	60 (78)

Numbers in brackets denote all hares released in a given year, while those without brackets relate to the number of animals that were radio-collared

### Radiotelemetry

Radiotelemetry was applied with a view to survival rates being monitored accurately, along with the causes of death of released hares. Tagging involved the affixing of collars with radio transmitters and internal antennae to the 60 released specimens. TXP-1 and TXP-R transmitters (Televilt, Sweden), especially designed for the hares, were used. The weight of radio transmitters was 40 and 55 g, respectively, which is below 5 % of the hare’s mean body mass. The radio transmitters in question operate at frequencies of 150,000–151,000 MHz, and the signal range indicated by the manufacturer is about 2 km. In practice, however, this proved to be very variable, and depended mainly on atmospheric conditions, as well as the season. Radio transmitters were supplied with so-called active mortality sensors that emitted a specific (more frequent) signal in the event of an animal’s death. The delay switch of mortality signal was approximately 4 h of complete immobility of the animal. If radio transmitter signal changed to mortality, as soon as possible we guided in the direction of the stronger signal without triangulation. Animals were located with the aid of a type RX-98 H receiver (also from Televilt), as well as a four-element external antenna of the Yagi type (Y-4FL). Hares were monitored over a 4-year period extending between November 2005 and November 2009. Different individuals were located with the aid of a traditional triangulation method (Mech 1983; Lenth 1981; Harris et al. 1990; Kenward 1993; Rühe 1999). This was used around two to three times a week (average of 2.8 ± 1.3 times), by both day and night. However, during the first 2 weeks after hares were released, they were located on a daily basis. Radiotelemetry provided a basis for the determination of survival rates among hares, as well as the rates and causes of mortality.

### Survival among released hares, as well as rates and causes of mortality

Survival and mortality rates among hares were analysed by reference to the time elapsing after release until losses occurred. Active sensors for mortality present in the collars with the radio transmitters provided for the rapid location of dead individuals. Causes of death were then determined as far as possible by careful examination of the retrieved dead hares, their remains, or the collars alone. The assessment of causes of death also took into account the place where the radio transmitter was discovered (e.g., close to a fox’s den or built-up area), as well as any tracks or spoor left in the vicinity. Furthermore, all cases of loss of radio contact (we cannot exclude a sporadic damage of radio transmitters as a reason of loss of contact) or non-discovery of a tagged hare (disappearance) in the first 12 months of radio-tracking were treated as losses. A similar procedure was followed when only the radio transmitter was found, and there was no possibility of determining the cause of death.

The date of death was considered to be that of the discovery of the dead individual or transmitter, or else that of the last radio contact. Imminent exhaustion of battery power on radio transmitters was heralded — about 10 days in advance — by a change in the radio signal emitted. The analysis of month-on-month changes in mortality and survival rates was served by the compilation of abbreviated life tables (Krebs 1997), in which reference is made to such parameters as:

*x*: age class
*l*_*x*_: number of individuals surviving through to the start of age class *x*
*d*_*x*_: number of individuals dying in the course of the age interval *x* to *x* + 1
*q*_*x*_: the specific mortality index (*d*_*x*_/*l*_*x*_)

### Statistical methods

The statistical processing of results made use of parametric tests, i.e., analysis of variance (ANOVA) and *t*-test, as well as a non-parametric test in the form of the Mann–Whitney *U*-test. Differences at *p* ≤ 0.05 were regarded as significant, and those achieving *p* ≤ 0.01 as highly significant.

## Results

### Survival among released hares

Among the 60 radio-collared hares involved in the successive releases, 22 survived for more than 12 months, which is to say that 38 individuals died in that period. Although animals died between days 1 and 243 after release, for those that did go on to die at some point in the first year, the mean periods survived — similar for females and males — were 64 (SD = 68) and 57 (SD = 81) days, respectively (*t*-test, *t* = 1.054, *p* = 0.298).

It was not possible to determine the mean longevity of all released hares, since contact was lost with the majority of individuals (*n* = 15) surviving longer than 12 months. This loss of contact did not denote death, but rather the exhaustion of batteries in radio transmitters. Among the total 60 radio-collared hares, 15 females (38 %) and seven males (33 %) could be located over periods exceeding 12 months, the difference in relation to gender not assuming statistical significance (*t*-test, *t* = 0.344, *p* = 0.734). On average, individuals that could still be located after 12 months could also be located for a further 7 months (range, 1–14 months). Mean duration of period over which radio contact was capable of being maintained with hares living longer than 12 months overall was 585 days (SD = 117; range 426–789) and 567 (SD = 112; range 374–677) for females and males, respectively.

Information on dates of loss of hares within the first 12 months of release made it possible for us to create the kind of abbreviated life table applied in population studies (Table 2). This was then used as a basis for analysis of month-on-month changes in rates of mortality and survival among individuals during their first year of life in the new environment. A specific index of mortality assuming values between 0 and 1 was also designated, this being used to identify months in which the greatest threat was posed to the released hares.

**Table 2:** Abbreviated life table for hares in the first year after release

*x*	*l*_*x*_	*d*_*x*_	*q*_*x*_
0 (between 25.11 and 01.01)	60	24	0.40
1 (between 01.01 and 01.02)	36	1	0.03
2 (between 01.02 and 01.03)	35	1	0.03
3 (between 01.03 and 01.04)	34	4	0.12
4 (between 01.04 and 01.05)	30	3	0.10
5 (between 01.05 and 01.06)	27	2	0.07
6 (between 01.06 and 01.07)	25	2	0.08
7 (between 01.07 and 01.08)	23	1	0.04
8 (between 01.08 and 01.09)	22	0	0.00
9 (between 01.09 and 01.10)	22	0	0.00
10 (between 01.10 and 01.11)	22	0	0.00
11 (between 01.11 and 01.12)	22	0	0.00

*x* denotes age class (months), *l*_*x*_ represents the no. of animals reaching the start of the given age class, *d*_*x*_ is the no. of animals dying in the given month, and *q*_*x*_ is the specific index of mortality (*d*_*x*_/*l*_*x*_))

### Mortality in the first 12 months after release

**Fig. 1:**
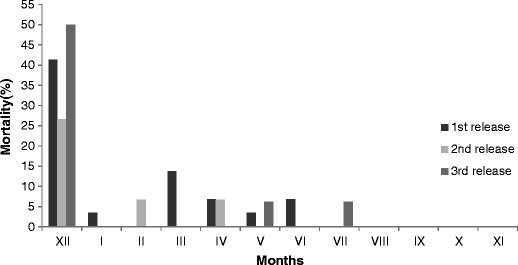
Mortality rates among hares in the first 12 months after releases that took place in November 2005, 2006 and 2007 in Central Poland

The overall mortality noted among hares during the first 12-month period after release was ca. 63 %. In consecutive years of release (2005, 2006 and 2007), rates were of 76 %, 47 % and 63 %, respectively (Fig. 1). The greatest mortality among released hares was observed in the very first month after release. This period saw about 40 % of all marked hares succumb (Fig. 1). The second period of enhanced mortality in turn coincided with the March–late August breeding period, during which monthly mortality was in the range of 0–7 %, equating to 20 % overall.

The highest first-month mortality was observed among hares released in 2007, which amounted to 50 % of all individuals radio-collared in that period (Fig. 1). The lowest figure was the ca. 27 % noted among individuals released in 2006. The highest breeding-season mortality (of ca. 30 %) was found among hares released in the first year of the study, while the lowest was ca. 7 % reported for animals released a year later in 2006 (Fig. 1). None of the three consecutive groups of hares released suffered any losses at all in the period from August through to the end of November.

In response to the high level of mortality characterising the first month after release, an analysis regarding the influence of body mass of released individuals on their longevity in the given period was carried out. This revealed no significant relationship between the parameter in question and the mortality rate among released hares in their first month at large (ANOVA, *F* = 0.06, *r* = −0.05, *n* = 23, *p* = 0.812).

### Causes of losses among released hares

Natural causes of death among released hares entailed predation by mammals (foxes, dogs and other [unidentified] species). Of the 24 fatalities in the first month following release, ca. 37 % were the result of predation, mostly by foxes. In other months, such events were sporadic in nature, to the extent that ca. 31 % of all losses occurring throughout the research period were of this kind (Table 3).

**Table 3:** Causes of death among hares released

Causes of death	No. of individuals	% of individuals
Foxes, dogs and other carnivores	14	31
Road kills	3	7
Poaching	6	13
Unidentified (collars found)	4	9
Disappeared	18	40
Total	45	100

Hares were only killed on roads during the breeding season, and these events represented nothing more than odd fatalities among the released animals. Confirmed cases of poaching accounted for around 13 % of incidences of death among introduced hares. In turn, in several cases all that was found was the radio-collar itself, with no possibility of identifying causative agent, or of determining the cause of death.

Besides the causes discussed previously, there were as many as 40 % of cases in which it proved impossible to determine the precise date and causes of losses (Table 3). This is to say that the loss of radio signal and disappearance of released individuals took place in unexplained circumstances. Since the majority of these cases concerned the first 12 months after release, it was possible to preclude breakdown or the exhaustion of batteries in radio transmitters as reasons. All cases of this kind were in fact classified as losses, with such individuals being regarded as absent from the study area (Table 3). In the first month after release, disappearance or poaching together accounted for as many as 50 % of all losses noted in the period.

## Discussion

Actions to resettle hares have earned for themselves a reputation for ultimate failure. Some researchers report that released or reintroduced individuals proved incapable of founding worthwhile, stabilised populations (Fiechter 1983, 1988; Pépin and Cargnelutti 1985, 1987; Marboutin et al. 1990; Angelici 1995; Riga et al. 1997). At a more refined level, there are data suggesting that survival rates and better adjustment to new environment is better among wild hares captured and that resettled to another place than among those released after having been raised in captivity (Fiechter 1983, 1988; Pépin and Cargnelutti 1985, 1987; Benmergui et al. 1990; Marboutin et al. 1990). However, in all experiments described above, the cage-reared hares were used as opposed to those derived from open-field enclosures practiced only in the last few years (Dziedzic et al. 2007).

At the same time, only a very few studies have been published on the effectiveness of introduction attempts for hares, and the way in which they organise spatially in their new environment. Only a very few ever employed radiotelemetry (except in a handful of cases like Marboutin et al.1990; Angelici et al. 1999, 2000), and the durations of monitoring periods have been limited to less than 12 months.

### Survivorship, mortality rates and causes of death among introduced hares

The survival rate in the first 12 months after release into the wild that we observed (37 %) is higher for hares originating from enclosure breeding than among the cage-raised individuals whose release is described in various other studies (e.g., Angelici et al. 2000). Our work showed that 22 of the radio-collared hares survived beyond the 12-month threshold. On average, radio contact was maintained with these animals for a further 7 months. Of course, the end of the capacity to locate individuals after that was more the result of the batteries in transmitters going flat than of death. This fact precluded the determination of actual mean or maximal life spans of introduced animals. What is clear, however, is that some animals released into the wild are able to survive in their new environment for longer than the life span of a battery (which is to say 18 months or so).

One of the few similar studies applying radiotelemetry and continued with for more than 1 year was that conducted by Angelici et al. (2000), although they used cage-raised hares. The results showed that all 44 released animals were dead within a year of release. Indeed, the mean further lengths of life for released females and males were similar, at around 67 and around 53 days, respectively. Likewise, the results presented here confirm that gender does not have any major effect in influencing survival.

The study by Angelici et al. (2000) had no information to offer on the survival of hares in successive months after release. The authors merely stated that, within the first month following release, survival rates for hares in their new environment was about 27 %. Beyond that, the survival period for remaining individuals in the course of the next 11 months was between 75 and 332 days. At the same time, they pointed out that the indicator for survival following release of captive-bred hares was of lower value than for wild specimens. This is also confirmed by research on wild hares netted from their natural environment, radio-collared and then released back at the same place. The rate of survival of such individuals was found to amount to about 67 % over a 3-month (January–March) period following release (Zaccaroni et al. 2009), or else about 70 % over a 5-month (May–September) period (Marboutin and Aebischer 1996). For comparison, the corresponding figures in the present study were of 57 % in the first 3 months after release, and 45 % at the end of the first 5 months. This point could suggest better adjustment to the new environment of enclosure-reared hares as compared with cage-reared ones; however, this rate is still lower when compared with wild individuals captured and released in the same place. Similar observations pointing to the greater efficacy of released measures involving animals from the natural environment as opposed to captive breeding were made by Fischer and Lindenmayer (2000), drawing on some several hundred studies devoted to this subject matter.

At the same time, the results of the present study confirm previous data attesting to maximum mortality among released hares in the first month after release. The research carried out by Angelici et al. (2000) shows this at a level in excess of 70 % among cage-reared hares. Where hares deriving from enclosure-based breeding are concerned, the results also indicate much more limited mortality (of around 40 %) in a period of the same length. Furthermore, the figure in question was never exceeded in subsequent months. Overall, it may be concluded that cage-reared hares are much less well adapted to the conditions of the natural environment than animals born and reared in large enclosures resembling an agricultural landscape. Nevertheless, in the first few days after release into a new environment, hares are always disorientated, and engage in a search for new places to take shelter and forage. By the same token, they face the greatest level of threat and danger in the period in question. Furthermore, inadequate preparation of the environment prior to the commencement of the release process (in situ protection) — which is to say a failure to reduce predator numbers, to offer supplementary feeding sites, to ensure shelter, to make adaptation enclosures available, as well as to engage in constant monitoring of the release areas — all combine together to reduce the chances of hares surviving the very first period of life in a new environment.

The results of our research also point to high mortality rates among released hares in the March–July period. The period in question is the breeding season for hares, and is thus associated with higher levels of activity and mobility both day and night. In these circumstances, animals most probably fall prey more easily to predators, but are furthermore at greater risk of being hit by cars or of facing other dangers as they move from place to place in search of a mate. Also needing to be stressed is the fact that overall mortality rates among wild hares (both adult and young) are high during the growing season, even reaching 72–74 % (Goszczyński and Wasilewski 1992; Juszko 2005).

Published results regarding causes of death among released hares point first and foremost to predation by mammals, mainly foxes (Angelici 1995; Riga et al. 1997; Angelici et al. 2000). The influence of this factor in reducing populations gained support from earlier studies on causes of death among hares (Goszczyński et al.^1976^; Pielowski and Raczyński 1976; Lindström et al. 1986; Reynolds and Tapper 1989, 1995a, b; Goszczyński and Wasilewski 1992; Spittler 1976; Panek and Kamieniarz 1999; Vaughan et al. 2003). In the study area, predation due to foxes, dogs or other unidentified carnivores accounted for some 31 % of all deaths among hares. When set against a figure in excess of 75 % noted by Angelici et al. (2000) for deaths arising among released hares, this factor can be thought to have had a relatively limited impact in this case. Nevertheless, similar results were obtained (for wild hares) by Juszko (2005), whose work in central Poland revealed a 32 % annual reduction in numbers attributable to foxes as key predators. Setting these data against the results of earlier research (i.e., the 18 % rate obtained by Goszczyński and Wasilewski 1992, and the 10 % reported by Pielowski 1979), we find clear confirmation of a relationship whereby the lower the population density of hares as prey, the higher the percentage reduction in the population foxes are capable of inducing. On the other hand, work carried out at the same time on changes in abundance and dietary composition in foxes pointed to the limited contribution of hares to the diet of the carnivore in terms of either frequency of kills or biomass. The density of foxes was average by Polish standards (Nikonowicz 2009), and it can be concluded that the overall decline in numbers of hares ensured their more limited representation in the diet of foxes, in line with numbers (Jędrzejewski and Jędrzejewska 2001; Panek 2007). At the same time, the carnivores are capable of playing a major role in reducing the number of hares, notwithstanding the limited contribution to their diet that such prey items actually make.

In this study, mortality caused by fox predation was mainly characteristic of the winter months, this linking up with data maintaining that foxes may play a key role in reducing numbers of wild hares in winter in particular, accounting for as much as 24 % of the number of individuals present in autumn (Juszko 2005).

Alongside foxes, other identified predators reducing numbers of introduced hares were vagrant dogs. Nationwide, cats and dogs not controlled in any way by people penetrate both field and forest areas, representing an ongoing, major problem apparently not noted on such a scale in other European countries. The causes of death noted for hares did not in this case extend to disease of any kind. Of course, it is impossible to preclude the idea that a diseased or infected animal succumbed and was then rapidly consumer by a predator.

The largest group of factors inducing losses among hares introduced into the study area fell within the so-called "unexplained circumstances." Bearing in mind the distances potentially coverable by hares, and noting the regularity with which fieldwork was carried out (2–3 times per week), the loss of radio contact with radio-collared hares and the total inability to recover either animal or equipment even when implausibly large areas were searched is quite indicative of illegal human activity, notably poaching of the animal and subsequent destruction of the transmitters. Further confirmation of this hypothesis was offered as acts of poaching were documented by the employees of the State Forests, the Hunting Guard or the Police. Together, these established cases accounted for 13 % of all recorded losses. In the first month after release, disappearance or poaching accounted for a total of 50 % of all losses noted in the period. By comparison, predator pressure accounted for around 37 % of losses. Similar work carried out in Western Europe (e.g., Marboutin and Aebischer 1996; Angelici et al. 2000) revealed only sporadic cases of the loss of radio contact or the disappearance of radio-collared individuals.

It is probable that poaching does have an influence on numbers of wild animals in Poland. In the case of hares, it may be a significant factor reducing numbers, especially where populations are anyway small. The work by Dziedzic et al. (2000) shows how the problem of poaching in Poland has intensified markedly in recent years, to the point where intense pressure is now being exerted. In the 1996/97 season alone, snares and other kinds of traps were discovered to contain some 4000 hares. Certainly, the results presented here allow it to be concluded that poaching may have a major negative influence on the efficacy of releasing or reintroduction efforts — and the subsequent chances of populations of hares rebuilding.

## References

[CR1] Angelici FM (1995) Ecologia della leper europea *Lepus europaeus* Pallas 1778 nel Pre-Appennino Laziale. Analisi di individui immessi per il ripopolamento venatorio. PhD thesis, University of Rome ‘La Sapienza’, 68 pp (In Italian)

[CR2] Angelici FM, Riga F, Boitani L, Luiselli L (1999). Use of dens by radiotracked brown hares *Lepus europaeus*. Behav Process.

[CR3] Angelici FM, Riga F, Boitani L, Luiselli L (2000). Fate of captive-reared brown hares *Lepus europaeus* released at a mountain site in central Italy. Wildl Biol.

[CR4] Benmergui M, Reitz F, Fiechter A (1990). Taux de reprise et dispersion de lièvres (*Lepus europaeus*) sauvages d’Europe centrale relâchés dans l’Est de la France. Gibier Faune Sauvage.

[CR5] Boye P (1996). Ist der Feldhase in Deutschland gefährdet?. Nat Landsch.

[CR6] Dziedzic R, Kamieniarz R, Majer-Dziedzic B, Wójcik B, Berger S, Flis M, OlszakK ŻM (2000). Przyczyny spadku liczebności zająca szaraka w Polsce.

[CR7] Dziedzic R, Błaszczyk J, Kołdyka L (2007) Restytucja zajęcy - hodowla kwaterowa -wsiedlanie. Nauka łowiectwu: część 2: Zającowi na ratunek, pp 114–118

[CR8] Edwards PJ, Fletcher MR, Berny P (2000). Review of the factors affecting the decline of the European brown hare, *Lepus europaeus* (Pallas, 1778) and the use of wildlife incident data to evaluate the significance of paraquat. Agric Ecosyst Environ.

[CR9] Fiechter A (1983). Premiers resultants de suivis télémetriques de levrauts de repeuplement. Bull Mensuel de l’Off National de la Chasse.

[CR10] Fiechter A (1988). Survie et dispersal de lièvres importés et de levrauts d’élevage lachés. Suppl Ricerche Biol Selvaggina.

[CR11] Fischer J, Lindenmayer DB (2000). An assessment of the published results of animal relocations. Biol Conserv.

[CR12] Frolich K, Meyer HD, Pielowski Z, Ronsholt L, von Secklanzendorf S, Stolte M (2003). European brown here syndrome in free-ranging hares in Poland. J Wildl Dis.

[CR13] Goszczyński J, Ryszkowski L, Truszkowski J, Pielowski Z, Pucek Z (1976). The role of European hare in diet of predators in cultivated field system. Ecology and management of European hare populations.

[CR14] Goszczyński J, Wasilewski M (1992). Predation of foxes on a hare population in central Poland. Acta Theriol.

[CR15] Harris S, Cresswell WJ, Forde PG, Trewhella WJ, Woollard T, Wray S (1990). Home range analysis using radio-tracking data – a review of problem and techniques particularly as applied to the study of mammals. Mammal Rev.

[CR16] Homolka M, Zima J, Mitchell-Jones AJ, Amori G, Bogdanowicz W, Kryštufek B, Reijnders PJH, Spitzenberger F, Stubbe M, Thissen JBM, Vohralík V, Zima J (1999). *Lepus europaeus*. The atlas of European mammals.

[CR17] Hutchings MR, Harris S (1996). The current status of brown hare (*Lepus europaeus*) in Britain.

[CR18] Jezierski W (2004). Zając, ginący gatunek. Łowiec Polski.

[CR19] Jędrzejewski W, Jędrzejewska B (2001). Ekologia zwierząt drapieżnych Puszczy Białowieskiej.

[CR20] Juszko S (2005) Wpływ drapieżnictwa na śmiertelność zająca szaraka w środkowej Polsce. Praca doktorska. Katedra Ochrony Lasu i Ekologii, Zakład Zoologii Leśnej i Łowiectwa. SGGW. Warszawa

[CR21] Kałuziński J, Pielowski Z, Pielowski Z, Pucek Z (1976). The effect of technical agriculture operation on the hare population. Ecology and Management of European hare population.

[CR22] Kenward R (1993). Wildlife radio tagging: equipment, field techniques and data analysis.

[CR23] Krebs CJ (1997) Ekologia. Wydawnictwo Naukowe PWN Warszawa, pp 159–184

[CR24] Lenth RL (1981). On finding the source of a signal. Technometrics.

[CR25] Lewandowski K, Nowakowski J (1993). Spatial distribution of Brown hare *Lepus europaeus* in habitats of various types of agriculture. Acta Theriol.

[CR26] Lindström E, Andrén H, Angelstam P, Widén P (1986). Influence of predators on hare population in Sweden: a critical review. Mammal Rev.

[CR27] Marboutin E, Benmergui M, Pradel R, Fiechter A (1990). Survival patterns in wild and captive reared leverets (*Lepus europaeus*, Pallas) determined by telemetry. Gibier Fauna Sauvage.

[CR28] Marboutin E, Péroux R (1995). Survival pattern of European hare in a decreasing population. J Appl Ecol.

[CR29] Marboutin E, Aebischer NJ (1996). Does harvesting arable crops influence the behaviour of the European hare *Lepus europaeus* ?. Wildl Biol.

[CR30] Marboutin E, Brey Y, Péroux R, Mauvy B, Lartiges A (2003). Population dynamics in European hare: breeding parameters and sustainable harvest rates. J Appl Ecol.

[CR31] Mech LD (1983). Handbook of animal radio-tracking.

[CR32] Misiorowska M, Wasilewski M (2008). Spatial organisation and mortality of released hares – preliminary results. Ann Zool Fenn.

[CR33] Nikonowicz Ł (2009) Rozmieszczenie, liczebność i skład pokarmu lisa (*Vulpes vulpes*) na terenie OHZ Garwolin. Praca magisterska. Katedra Ochrony Lasu i Ekologii, Zakład Zoologii Leśnej i Łowiectwa. SGGW, Warszawa

[CR34] Ninov N (1990). Der Einfluss einiger ökologischer Faktoren auf die Dynamik der Hasenbesätze in Bulgarien. Beitr Jagd-und Wildforsch.

[CR35] Panek M, Kamieniarz R (1999). Relationships between density of brown hare *Lepus europaeus* and landscape structure in Poland in the years 1981–1995. Acta Theriol.

[CR36] Panek M (2007). Redukcja lisów a sytuacja zajęcy. Łowiec Polski.

[CR37] Pépin D, Cargnelutti B (1985). Dispersal et cantonnement de lièvres de repeuplement (*Lepus europaeus*). Biol Behav.

[CR38] Pépin D, Cargnelutti B (1987). Développement de strategies d’utilisation de l’espace et du temps lors de l’implantation en nature de leveauts (*Lepus europaeus*) issus d’élevage. Gibier Faune Sauvage.

[CR39] Pfister H, Kohli L, Kästili P, Birrer S (2002) Feldhase. Schlussbericht 1991–2000. Bundesamt für Umwelt, Wald und Landschaft, BUWAL(eds), Schriftenreihe Umwelt 334, Berlin

[CR40] Pielowski Z, Raczyński J (1976) Ecological conditions and rational management of hare population. In Pielowski Z, Pucek Z (eds) Ecology and management of European hare populations. Państwowe Wydawnictwo Rolnicze i Leśne, Warszawa, pp 269–286

[CR41] Pielowski Z (1979). Zając.

[CR42] Rattenborg E (1991). Climatic Influence on Fluctuations in the Danish Hare Bag 1941–1988.

[CR43] Reichline T, Klansek E, Hakländer K (2006). Diet selection by hares (*Lepus europaeus*) in arable land and its implications for habitat management. Eur J Wildl Res.

[CR44] Reynolds J, Tapper S (1989) Foxes and hares. A full report of the research and other activities of the Game Conservancy during the 1988. The Game Conservancy, Fordingbridge, pp 98–101

[CR45] Reynolds J, Tapper S (1995). The ecology of red fox *Vulpes vulpes* in relation to small game in rural southern England. Wildl Biol.

[CR46] Reynolds J, Tapper S (1995). Predation by foxes *Vulpes vulpes* on brown hares *Lepus europaeus* in central southern England, and its potential impact on annual population growth. Wildl Biol.

[CR47] Riga F, Boitani L, Caporioni M, Fioramonti L, Gemma F, Laurenti A, Angelici FM (1997). Esito dei ripopolamendi di Lepre europea (*Lepus europaeus*) in un’area del Preappennino laziale. Suppl Ricerche Biol Selvaggina.

[CR48] Roedenbeck IA, Voser P (2008). Effects of road on spatial distribution, abundance and mortality of brown hare (*Lepus europaeus*) In Switzerland. Eur J Wildl Res.

[CR49] Rühe F (1999). Effect of standard structures in arable crops on brown hare (*Lepus europaeus*) distribution. Gibier Faune Sauvage.

[CR50] Santilli F, Galardi L (2006). Factors affecting brown hare (*Lepus europaeus*) hunting bags in Tuscany region (Centarl Italy). Hystrix - It J Mammal (n.s.).

[CR51] Schmidt KR, Jennings NV, Robinson A, Harris S (2004). Conservation of European hares *Lepus europaeus* in Britain: is increasing habitat heterogeneity in farmland the answer?. J Appl Ecol.

[CR52] Slamečka J, Csànyi S, Erhaf J (1991). The influence of ecological arrangements on brown hare population. XXth Congress of the International Union of Game Biologist.

[CR53] Slamečka J, Hell P, Jurcik R (1997). Brown hare in the west Slovak lowland. Acta Sci Nat Brno.

[CR54] Smith RK, Jennings NV, Harris S (2005). A quantitative analysis of the abundance and demography of European hares *Lepus europaeus* in relation to habitat type, intensity of agriculture and climate. Mammal Rev.

[CR55] Spittler H, Pielowski Z, Pucek Z (1976). Witterungsfaktoren als Grundlage für Vorhersagen über die Entwicklung des Hasenbesatzes. Ecology and management of European hare populations.

[CR56] Tapper S (1995) The status of brown hare (*Lepus europaeus*) in Britain. In: Hare, International Symposium, Czempiń 1992, pp 354–359

[CR57] Van Wieren SE, Wiersma M, Prins HHT (2006). Climate factors affecting a brown hare (*Lepus europaeus*) population. Lutra.

[CR58] Vaughan N, Lucas EA, Harris S, White PC (2003). Habitat association of European hares *Lepus europaeus* in England and Wales: implications for farmland management. J Appl Ecol.

[CR59] Vuković M, Tvrtković N (2006) Zec, brown hare, *Lepus europaeus* Pallas, 1778. U: Tvrtković N. (ur): Crvena knjiga sisavaca Hrvatske. Ministrastvo culture, Državni za zaštitu prirode, Zagreb, 2006, pp 100–101

[CR60] Zaccaroni M, Biliotti N, Calieri S, Ferretti M, Genghini M, Riga F, Trocchi V, Dessi-Fulgheri R (2009) Habitat use by brown hares (*Lepus europaeus*) in an agricultural ecosystem in Tuscany (Italy) using GPS collars: implication for agrienvoronmental management. Short communication, published in the act of the conference, IUGB 2009

